# *TTR* Gly83Arg Mutation: Beyond Familial Vitreous Amyloidosis

**DOI:** 10.3389/fneur.2021.821003

**Published:** 2022-02-03

**Authors:** Zhenxian Li, Kang Du, Xujun Chu, He Lv, Wei Zhang, Zhaoxia Wang, Yun Yuan, Lingchao Meng

**Affiliations:** ^1^Department of Neurology, Peking University First Hospital, Beijing, China; ^2^Beijing Key Laboratory of Neurovascular Disease Discovery, Beijing, China

**Keywords:** transthyretin amyloidosis, Gly83Arg, China, ocular symptoms, extraocular symptoms

## Abstract

**Background:**

Gly83Arg variation is a type of *TTR* mutation specific to the Chinese population. Patients of hereditary transthyretin amyloidosis (ATTR) with Gly83Arg variation predominantly present with blurred vision and most of these cases are reported by ophthalmologists. There is currently no systematic assessment of extraocular features of ATTR with Gly83Arg variation.

**Methods:**

Six patients and two asymptomatic carriers with molecularly confirmed Gly83Arg variation of ATTR from three unrelated families were identified by sequencing the *TTR* gene. The clinical, electrophysiological, ultrasonic, and pathological data were collected and analyzed.

**Results:**

This study included six patients and two carriers with *TTR* Gly83Arg mutation, all of whom came from the Han nationality of China. The average age of onset for the six patients was 39 years, and the course of disease ranged from 5 to 19 years. All the patients started with blurred vision, which was diagnosed as vitreous opacity (VO). Most of the patients developed sensory-motor polyneuropathies over years or even more than a decade (4–15 years) after VO. However, the heterogeneity of peripheral neuropathies among these patients remained large between families. Autonomic impairment also occurred after VO, with varying degrees of abnormalities seen in the associated autonomic assessments. None of the patients had any symptoms of cardiac impairment, but abnormal results were found in examinations. A combined biopsy of the sural nerve and muscle was also performed. Nerve pathology revealed the moderately reduced myelinated nerve fiber density and muscle pathology showed predominant neurogenic impairment accompanied by possible myogenic impairment.

**Conclusions:**

This is a detailed account of Gly83Arg mutation-related ATTR, focusing on the extraocular presentations of this special variant in Chinese. Clinical features of this variant are early-onset, ocular involvement predominance, neurological, and cardiac involvement along with the disease, and relatively long survival.

## Introduction

Hereditary amyloidosis is a group of genetic diseases characterized by tissue deposition of insoluble proteins and fibril aggregates, causing disorders involving different organs. Among the precursor proteins of amyloid, transthyretin (TTR) is common and devastating. Hereditary transthyretin amyloidosis (ATTR), caused by *TTR* gene mutations, is characterized by a length-dependent polyneuropathy and autonomic dysfunction, with multisystem involvement, namely, the heart, eyes, and kidney (1). More than 140 different point mutations have been identified in the *TTR* gene (2).

Gly83Arg, a unique *TTR* gene mutation in the Chinese population, is characterized by ocular involvement, which has been discovered and reported by ophthalmologists in recent years. Usually, it is considered as a mutation, causing familial vitreous amyloidosis (3–9). However, manifestations of the peripheral nerve system, autonomic nerve system, and the involvement of the heart and other organs are rarely reported. This study is intended to evaluate the extraocular features of our patients with Gly83Arg mutation and characterize the phenotype of this variation.

## Materials and Methods

### Participants

Six patients and two asymptomatic carriers from three unrelated families who were diagnosed with Gly83Arg variation of ATTR at the Department of Neurology, Peking University First Hospital, between September 2019 and April 2021 were included in this study. The detailed data on symptoms and signs were collected, namely, intact medical history, results of general medical examination, Neuropathy Impairment Score (NIS), Norfolk Quality of Life-Diabetic Neuropathy Score (Norfolk QOL), and Composite Autonomic Symptom Score-31 (COMPASS-31). Modified body mass index (mBMI) was calculated (BMI × serum albumin level). All the patients provided their informed consent for this study, and ethical approval was obtained from the Human Research Ethics Committee of the Peking University First Hospital.

### Laboratory Testing

The nerve conduction study (NCS) was performed to evaluate the involvement of the peripheral nerve system. A head-up tilt test and sympathetic skin response (SSR) were conducted to assess autonomic dysfunction. Ultrasonic cardiogram (UCG) and N-terminal probrain natriuretic peptide (NT-pro-BNP) were used to determine cardiac involvement.

### Ultrasonography

All the subjects underwent peripheral nerve ultrasound using the Philips Imaging System (iU Elite, Bothell, WA, USA) that measured and recorded the bilateral median, ulnar, sciatic, tibial, common peroneal, and sural nerves. To be more specific, the 17 MHz high-frequency linear array probe was used for the superficial nerves, namely, the median nerves, ulnar nerves, common peroneal nerves, and sural nerves, while the 9 MHz linear array probe was used for the deeper nerves, namely, the sciatic nerves and tibial nerves. The cross-sectional areas (CSAs) at the predetermined sites of each nerve were measured by tracing just inside the hyperechoic rim of the nerve. Thirty predetermined sites were measured of all the nerves according to the previously reported protocol (10).

### Pathology

A combined biopsy of sural nerve and gastrocnemius was performed in patients II-4 and III-6. One specimen of the sural nerve was fixed in 4% formaldehyde, paraffin-embedded, 8-μm sections, and stained with H&E, Congo-red, and Luxol fast blue. TTR immunohistochemical staining was also performed by standard techniques (DAKO). One specimen was fixed in 3% glutaraldehyde, postfixed in 1% osmium tetroxide, dehydrated through acetone, and embedded in Epon 812. Semithin sections for light microscopy were stained with toluidine blue. The muscle specimens were frozen in isopentane and cooled in liquid nitrogen. Serial frozen sections were stained using routine histological and histochemical methods, and immunohistochemical techniques for TTR (DAKO).

### Data Availability

Anonymized data will be shared by request from any qualified investigator.

## Results

### Clinical Features

**Figure 1:**
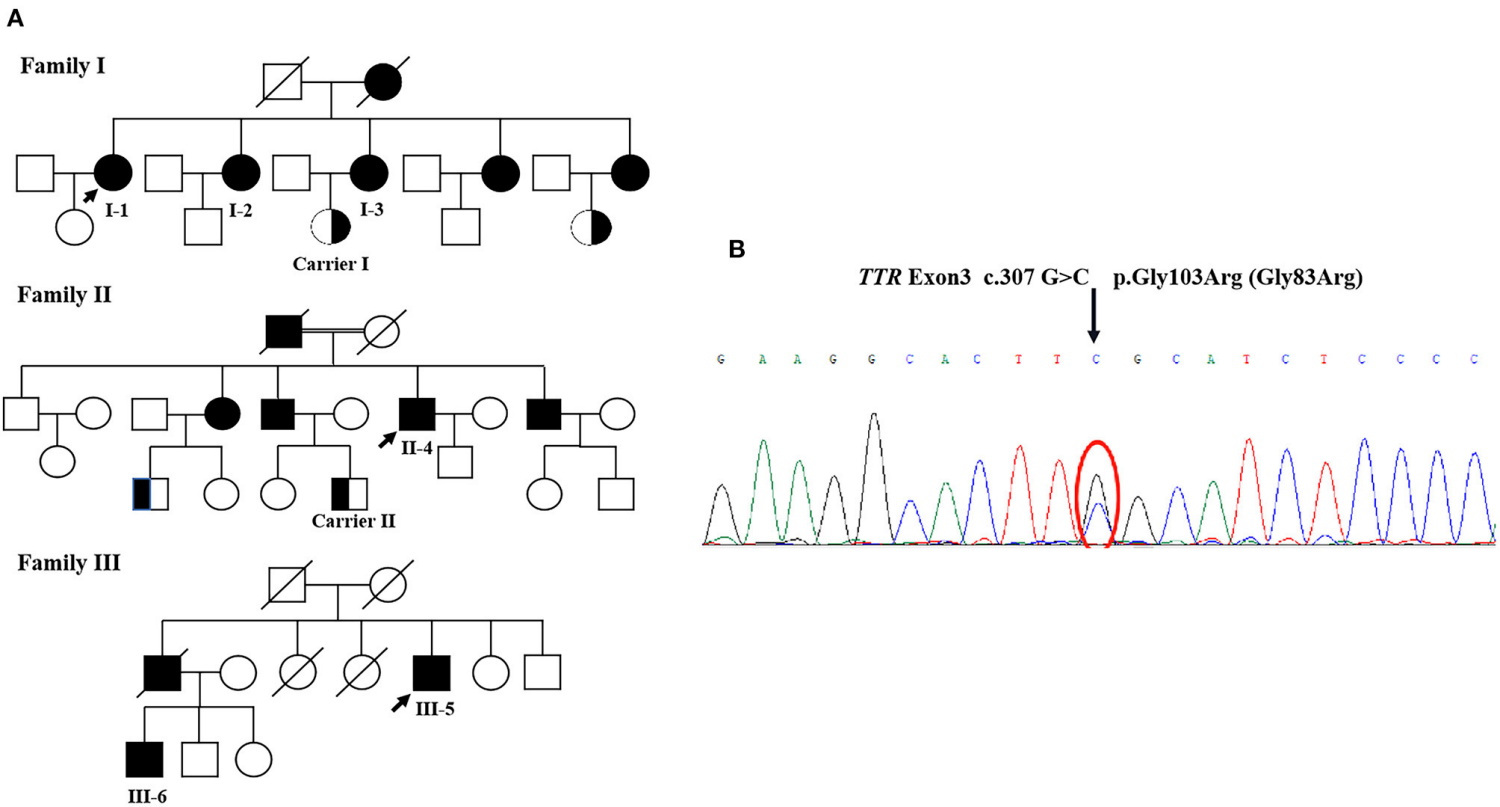
**(A)** The pedigree of three families. **(B)** The *TTR* sequencing results. Sanger sequencing of exons showed a G to C transversion (arrow), resulting in the substitution of glycin-103 by arginine (p.Gly103Arg, Gly83Arg).

All the six patients were of Chinese Han nationality, including three females (I-1,2,3) and three males (II-4, III-5,6), with the pathogenic variation of *TTR* c.307G>C, p.Gly103Arg (Gly83Arg) (Figure 1). The mean age at onset was 35 years for female patients and 43 years for male patients, with a total mean age at onset of 39 years (ranging from 34 to 44 years) and a total mean course of the disease of 12 years (ranging from 5 to 19 years). All six patients presented with ocular symptoms initially, mainly manifested as alternating and recurrent blurred vision in both eyes. All the patients underwent vitrectomy at least once except III-6. Extraocular symptoms were characterized by the peripheral nervous system and autonomic nervous system involvement. After the onset of ocular symptoms, three patients (I-1,2,3) developed intermittent numbness in both hands due to carpal tunnel syndrome (CTS), but only I-1 had sensory-motor polyneuropathy in 15 years after the eyes involvement. However, the three patients of the other two families (II-4 and III-5,6) started with numbness in lower limbs and then progressed to numbness of upper limbs and weakness of all extremities in 3–11 years after blurred vision. The neuropathies were far more severe than those of patients I-1,2,3, with higher NISs and Norfolk QOL scores than those of patients I-1,2,3. The three patients of I-1,2,3 had very mild autonomic dysfunction including dilute stool. The remaining patients (II-4 and III-5,6) suffered from varying degrees of diarrhea, constipation, impotence, dysuria, and skin color changes. Some of them had postural dizziness, dry mouth, or dry eyes. Autonomic symptoms were more severe for II-4 and III-6 than for others, with COMPASS-31 scores substantially higher than those of other patients. None of the six patients experienced cardiac symptoms so far. However, evidence of cardiac hypertrophy was observed in two of the six (II-4 and III-6) from the UCG test. Patient I-1 had mildly elevated NT-pro-BNP without abnormality in the UCG. Some patients had different degrees of weight loss, with relatively lower mBMI values compared with normal values. None of the above was found in either of the two carriers (Tables 1, 2). Only patient II-4 took tafamidis 20 mg orally once daily, while the other patients received no disease-modifying therapies.

**Table 1:** Characteristics of patients with symptomatic Gly83Arg-related ATTR in this study and reviews.

**Family-Patient**	**Sex**	**AO (years)**	**Course of disease (years)**	**Family history**	**Initial symptoms**	**Peripheral neuropathy**	**Autonomic neuropathy**	**Other abnormalities**	**Interval of blurred vision to SMPN (years)**	**References**
I-1	F	35	19	Positive	Blurred vision	CTS for 13 years, SMPN for 4 years	Intermittent dilute stool	BW loss	15	This study
I-2	F	34	17	Positive	Blurred vision	Mild CTS for 4 years	Intermittent dilute stool	BW loss	No SMPN	This study
I-3	F	36	10	Positive	Blurred vision	CTS for 10 years	Dilute stool for a long time	BW loss	No SMPN	This study
II-4	M	42	7	Positive	Blurred vision	SMPN for 4 years	ADC, dysuria, xerostomia, erectile dysfunction, skin color changes	Cardiac hypertrophy, BW loss	3	This study
III-5	M	43	12	Positive	Blurred vision	SMPN for 1 year	Constipation, dysuria, erectile dysfunction, skin color changes	BW loss	11	This study
III-6	M	42	5	Positive	Blurred vision	SMPN for 1 year	Diarrhea, dysuria, xerostomia, xerophthalmia, erectile dysfunction, skin color changes, postural dizziness	Cardiac hypertrophy, BW loss	4	This study
Family 1	M	41	3	Positive	Blurred vision	NA	NA	NA	NA	Chen et al. 2008 (3)
Family 2	M	41	3	Positive	Blurred vision	NA	NA	NA	NA	Chen et al. 2011 (11)
Family 3	M	43	1.5	Positive	Blurred vision	NA	NA	NA	NA	
Family 4	NA (n=3)	about 40	NA	Positive	Blurred vision	NA	NA	NA	NA	Xie et al. 2012 (4)
Family 5	M (n=4), F (n=4)	M 46 (n=4), F 50 (n=4)	NA	Positive	Blurred vision	NA	NA	NA	NA	Xie et al. 2013 (12)
Family 6	M (n=5), F (n=3)	46 (range 40-56)	NA	Positive	Blurred vision	NA	NA	NA	NA	Xu et al. 2013 (5)
Family 7	F	38	10	Positive	Blurred vision	NA	NA	NA	NA	Xie et al. 2016 (13)
Family 8	M (n=3), F (n=2)	M 32.3 (range 30-34); F 36.5 (range 34-39)	NA	Positive	Blurred vision	NA	NA	NA	NA	Zhang et al. 2013 (6)
Family 9	F (n=2)	42.5 (range 40-45)	NA	Positive	Blurred vision	NA	NA	NA	NA	
Family 10	M (n=2)	40 (range 40-40)	NA	Positive	Blurred vision	NA	NA	NA	NA	
Family 11	M (n=6), F (n=6)	M 41.8 (range 38-47); F 40.7 (range 37-43)	NA	Positive	Blurred vision (n=11), UL paresthesia (n = 1)	UL neuropathy in 10 cases, LL neuropathy in 4 cases at visit	NA	Cardiac hypertrophy in one case	NA	Liu et al. 2014 (7)
Family 12	F (n=2)	35 (range 35-35)	5, 3	Positive	Blurred vision	NA	NA	NA	NA	Yin et al. 2014 (8)
Family 13	F	39	8	Positive	Blurred vision	NA	NA	NA	NA	Xie et al. 2017 (9)

*ADC, alternation of diarrhea and constipation; AO, age of onset; BW, body weight; CTS, carpal tunnel syndrome; F, female; LL, lower limbs; M, male; NA, not available; SMPN, sensory-motor polyneuropathy; UL, upper limbs*.

**Table 2:** Extraocular characteristics of patients with symptomatic ATTR having the Gly83Arg variant.

**Family-Patient**	**Peripheral neuropathy**			**Autonomic dysfunction**		**Cardiac involvements**				**Clinical evaluation score**			
	**Nerve ultrasound**	**Sural nerve biopsy**	**Biopsy of gastrocnemius muscle**	**SSR**	**Head-up tilt test**	**NT-pro-BNP (pg/ml) (NV: <125 pg/ml)**	**IVS (mm) (NV:≤11 mm)**	**LVPW (mm) (NV:≤11 mm)**	**LVEF (%) (NV:≥50%)**	**NIS**	**Norfolk QOL score**	**COMPASS-31 score**	**mBMI (Kg/m^**2**^) × (g/L) (NV¶: 957.9–1,440.1)**
I-1	Proximal thickness and carpal canal thickness	ND	ND	Not recorded	ND	178	10	9.7	63.6	22	13	12.6	974
I-2	Proximal thickness	ND	ND	Normal	ND	96	11	11	76	6	11	10.4	965.6
I-3	Proximal thickness and carpal canal thickness	ND	ND	Normal	Negative	78	9.8	10	73.8	10	11	7.8	843
II-4	Proximal thickness and carpal canal thickness	Moderately reduced fiber density, predominant with small myelinated nerve fibers	Neuropathic pattern companied with possible myopathic impairment	Normal	Positive	74	12	12	65	81	48	27.12	983.4
III-5	Proximal thickness and carpal canal thickness	ND	ND	ND	Negative	52	7.4	8.2	71	54	80	10.00	888.30
III-6	Proximal thickness and carpal canal thickness	Moderately reduced fiber density	Neuropathic pattern companied with possible myopathic impairment	ND	Positive	166	13	14	43.7	44	79	61.18	858.3

*CTS, carpal tunnel syndrome; COMPASS-31, composite autonomic symptom Score 31; IVS, interventricular septal; LVEF, left ventricular ejection fraction; LVPW, left ventricular posterior wall; mBMI, modified body mass index; NCS, nerve conduction study; ND, not done; NV, normal value; Norfolk QOL score, norfolk quality of life-diabetic neuropathy score; NIS, neuropathy impairment score; SMPN, sensory-motor polyneuropathy; SSR, sympathetic skin response*.

¶ *: Referring to the normal values of previous literature (14)*.

### Electrophysiology

The six patients and two carriers completed neurophysiological examinations, namely, sensory nerve conduction from the bilateral median, ulnar, superficial peroneal, and sural nerves, and motor nerve conduction from the bilateral median, ulnar, common peroneal, and tibial nerves (partial data for patient III-5 were not available).

For patients I-1,3, the amplitudes of the sensory nerve action potential of bilateral median and ulnar nerves were not recorded. Patient I-2 had only a slight decrease in sensory conduction velocity of median and ulnar nerves. For patient I-1, the amplitude of the compound muscle action potential of the median nerve was also moderately reduced. Sensory nerve conduction in the lower extremities of patient I-1 was also impaired, but there were no electrophysiologic abnormalities in lower limbs for patients I-2,3. Patient III-6 had a relatively mild abnormality. The other two patients had more obvious electrophysiological abnormalities, indicating the axonal sensory-motor polyneuropathies (Table 3).

**Table 3:** Neuroelectrophysiological features of patients with symptomatic ATTR having the Gly83Arg variant.

**Nerves**	**Parameters**	**I-1**	**I-2**	**I-3**	**II-4**	**III-5**	**III-6**	**Normal values**
Median motor nerves	DML, ms	**6.6**	3.9	**4.5**	**6.1**	3.1	3.5	<4
	CMAP, mV	**2.9**	10.4	7.9	**1.9**	**4.2**	7.1	>5
	MCV, m/s	51.2	59.5	54.3	**47.5**	52.0	**49.7**	>50
Ulnar motor nerves	DML, ms	2.8	2.7	2.7	**3.7**	2.7	2.7	<3
	CMAP, mV	6.5	9.4	7.9	**4.0**	10.6	9.7	>4
	MCV, m/s	52.4	57.8	50.5	**41.7**	57.8	52.0	>50
Peroneal nerves	DML, ms	4.5	4.1	4.5	**6.6**	**NR**	3.7	<5.3
	CMAP, mV	3.9	4.5	5.6	**0.2**	**NR**	**2.0**	>2
	MCV, m/s	45.4	49.3	45.5	**34.9**	**NR**	**38.9**	>40
Tibial nerves	DML, ms	3.7	4.0	3.7	**6.9**	**NR**	4.9	<5
	CMAP, mV	4.7	7.8	10.2	**0.2**	**NR**	**3.2**	>3.5
	MCV, m/s	42.4	48.6	47.0	**38.2**	**NR**	40.1	>40
Median sensory nerves	SNAP, μV	**NR**	**5.0**	**NR**	**NR**	**3.0**	5.8	>5
	SCV, m/s	**NR**	**34.3**	**NR**	**NR**	**46.8**	**49.4**	>50
Ulnar sensory nerves	SNAP, μV	**NR**	8.9	**NR**	**NR**	10.6	5.2	>3
	SCV, m/s	**NR**	**48.2**	**NR**	**NR**	57.8	**47.6**	>50
Superficial peroneal nerves	SNAP, μV	**1.9**	8.1	6.3	**NR**	ND	13.0	>1
	SCV, m/s	**38.8**	52.0	47.3	**NR**	ND	49.2	>40
Sural nerves	SNAP, μV	**NR**	6.3	5.0	**NR**	ND	6.0	>1
	SCV, m/s	**NR**	51.5	48.9	**NR**	ND	41.1	>40

*CMAP, compound muscle action potential; DML, distal motor latency; MCV, motor conduction velocity; ND, not done; NR, not record; SCV, sensory conduction velocity; SNAP, sensory nerve action potential*.

*The SNAP and SCV of left ulnar sensory nerve in I-3 patient and right sural nerve in I-1 patient were not recorded*.

*The abnormal values were printed in bold*.

No abnormality was found in electrophysiological examinations of the two carriers.

### Ultrasonography

**Figure 2:**
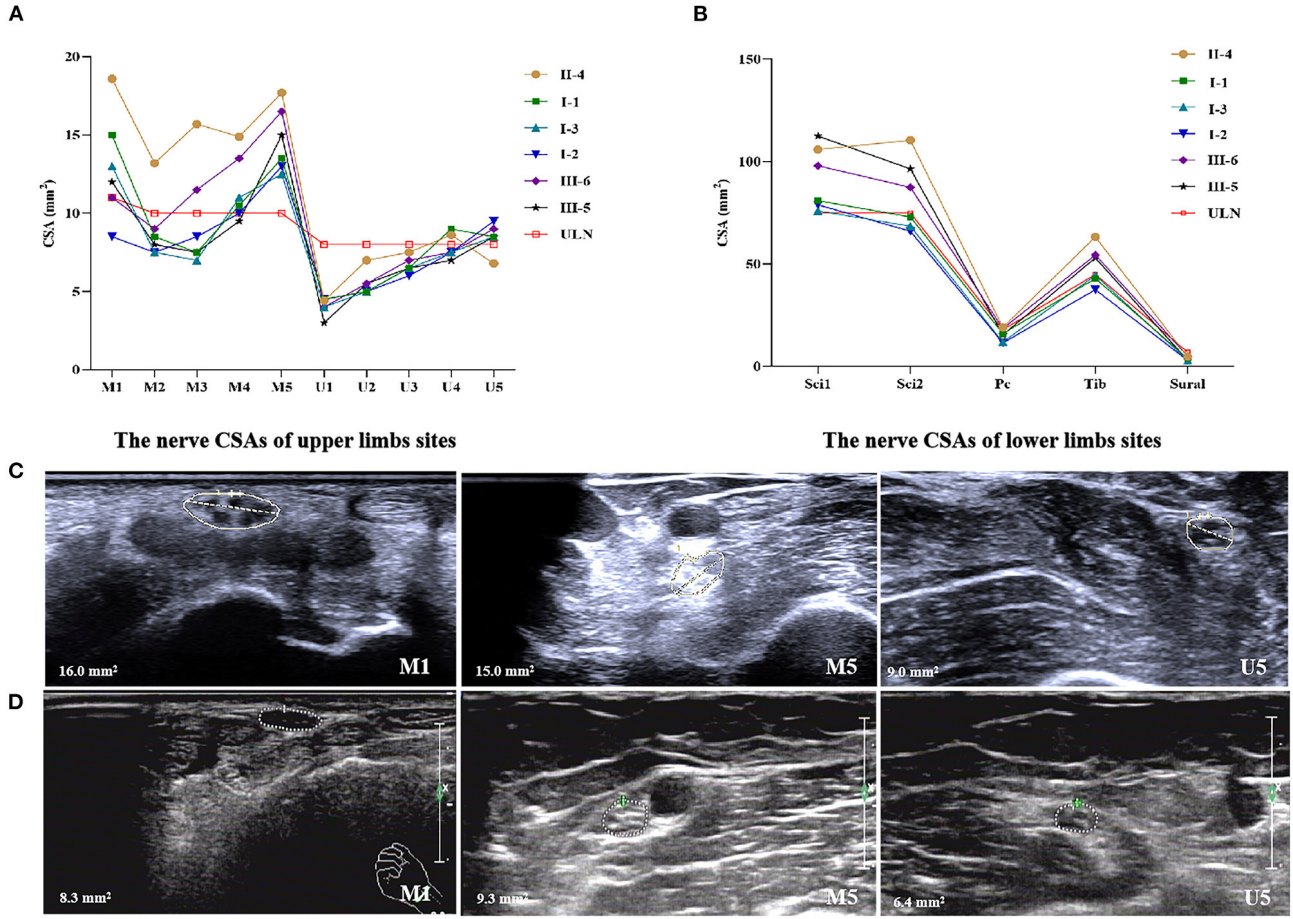
Nerve ultrasonography of patients with Gly83Arg mutation-related ATTR. **(A)** The nerve CSAs of upper limbs sites. **(B)** The nerve CSAs of lower limbs sites. **(C)** Enlargement of CSAs at the site of M1, M5, and U5 of patient III-5. **(D)** The same sites of healthy control. ULN, upper limit of normal in our center. (1) 10 sites that were measured in median nerves: M1, wrist (entrance to the carpal tunnel at the pisiform bone level); M2, distal forearm (the nerve reached the deep flexor digitorum and started to traverse between the deep flexor digitorum and the flexor pollicis longus); M3, proximal forearm (the clearest point before the nerve entered pronator teres); M4, elbow (elbow socket); M5, upper arm (from cubital fossa to the middle of the armpit). (2) Ten sites of ulnar nerves: U1, wrist (Guyon tube: between the pisiform bone and ulnar artery); U2, distal forearm (before the ulnar nerve branches off); U3, proximal forearm (2/3 between the wrist and elbow); U4, elbow (at the medial epicondyle of humerus); U5, upper arm (from cubital fossa to the middle of the armpit). (3) Four sites of sciatic nerves: Sci1, middle thigh; Sci2, 1/3 of mid-lower part of the thigh (before sciatic nerves were divided into common peroneal nerves and tibial nerves). (4) Two sites of tibial nerves: Tib, popliteal fossa (just after the tibial nerves were branched off by sciatic nerves). (5) Two sites of common peroneal nerves: Pc, capitulum fibulae. (6) Two sites of sural nerves: sural, lower 1/4 of the lower leg near lateral malleolus.

All the patients and two carriers completed nerve ultrasound examinations as in our previous study (10), including successive scans of the median and ulnar nerves in the upper limbs, the sciatic, tibial, common peroneal, and sural nerves in the lower limbs. The results revealed proximal median nerve (upper arm segment) enlargements in all the patients, with an average CSA of 14.7 mm^2^ (ranging from 12.5 to 17.7 mm^2^) compared with the upper limit of normal (ULN) of our center (10 mm^2^). Median nerve carpal thickening or reaching the ULN occurred in five of the six patients, with an average CSA of 13.0 mm^2^ (ranging from 8.5 to 18.6 mm^2^). Proximal ulnar nerve thickening occurred in five of the six patients, with an average CSA of 8.5 mm^2^ (ranging from 6.8 to 9.5 mm^2^). Proximal sciatic nerve thickening of the lower limbs occurred in all the patients, with an average CSA of 92.1 mm^2^ (ranging from 76.0 to 112.5 mm^2^). In patients II-4, III-5, and III-6, apart from nerve enlargements at the proximal sites of upper limbs, the nerve CSAs at the distal site of sciatic (Sci 2), common peroneal and tibial nerves of the lower extremities were also increased, with an average CSA of 83.7 mm^2^ (ranging from 66.0 to 110.5 mm^2^), 15.4 mm^2^ (ranging from 11.5 to 19.1 mm^2^), and 49.3 mm^2^ (ranging from 37.5 to 63.3 mm^2^), respectively (Figure 2).

For the two asymptomatic carriers, carrier-II had a slight enlargement of median nerves at proximal sites with 13.1 and 11.6 mm^2^ on the left and right sides. Carrier-I had normal nerve CSAs.

### Autonomic Function Tests

All patients performed autonomic function valuations, namely, head-up tilt test (four patients and one carrier) and SSR (four patients and two carriers). All patients performed the head-up tilt test except I-1,2. Orthostatic hypotension was observed in patient III-6 by the head-up tilt test, with obvious complaints of postural dizziness. Patient II-4 had no symptoms of postural dizziness, but the head-up tilt test was also positive, showing the subclinical orthostatic hypotension (Table 2). Patients I-1,2,3 and II-4 performed SSR examination, and only patient I-1 had the abnormality. Carrier-1 had a positive head-up tilt test result with the diagnosis of postural tachycardia syndrome. However, Carrier-1's SSR results of both hands and feet were normal.

### Pathology

**Figure 3:**
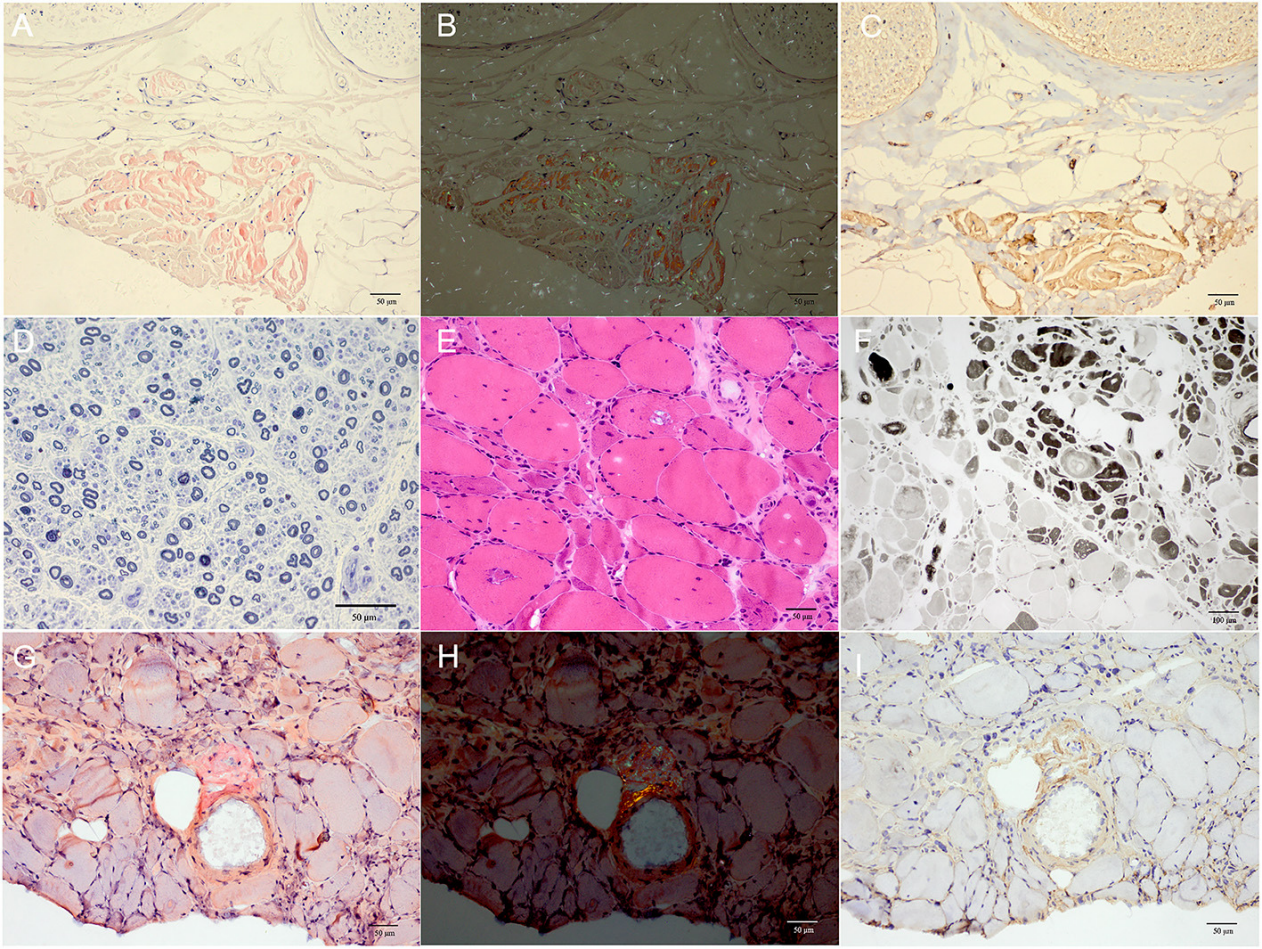
**(A–D)** Sural nerve biopsy of patient II-4. **(E–I)** Gastrocnemius muscle biopsy of patient III-6. **(A)** Many red deposits were found in the epineurial interstitial tissues by Congo-red staining. **(B)** The “apple-green” deposits were observed under polarized light. **(C)** The existence of TTR-related amyloid deposits was further proved by TTR immunohistochemical staining. **(D)** The moderately reduced myelinated fiber density with 4,175.7/mm^2^ of myelinated nerve fibers density and some axonal degeneration were observed with semithin section by toluidine blue staining. **(E,F)** Muscle biopsies showed both neurogenic changes (groups of small angular atrophic muscle fibers, involving two types) and myopathic changes (vacuoles muscle fibers) with H&E staining **(E)** and ATPase 4.6 staining **(F)**. **(G,H)** The Congo-red positive materials were also observed in the perivascular area with “apple-green” under polarized light and were positive by TTR immunohistochemical staining at the same site **(I)**. Scale bars = 50 μm in **(A–E,G–I)**; 100 μm in **(F)**.

Both patient II-4 and patient III-6 underwent nerve and muscle biopsy. The sural nerve pathology revealed moderately reduced myelinated nerve fiber densities from the semithin section. Axonal degeneration was observed. There were some thickened myelin sheath and obvious thickening of the basement membrane of some capillaries. There were no onion bulbs of myelinated nerve fibers observed. In patient II-4, many abnormal materials were found in the interstitial tissues in the epineurium and the existence of TTR-related amyloid deposits was proved by Congo-red staining, polarized light with “apple-green” and TTR immunohistochemical staining. In patient III-6, there was no positive material found by Congo-red staining in the sural nerve (Figure 3).

The biopsies of gastrocnemius muscle were also performed. H&E staining showed some necrotic muscle fibers with inflammatory cells infiltration and some regenerated muscle fibers in patient II-4, and muscle fibers with vacuoles in patient III-6. The fibers of angular atrophy were distributed in groups, involving two types, which were seen in adenosine triphosphatase (ATPase) staining, and typical target-like muscle fibers with nicotinamide adenine dinucleotide tetrazolium reductase staining were found in both patients, so were amyloid deposits in perifascicular interstitial regions and perivascular areas using Congo-red and TTR immunohistochemical staining (Figure 3).

### A Review of Clinical Features of Gly83Arg Patients Reported in China

In 2008, Chen et al. reported the first patient with Gly83Arg mutation of *TTR* gene in China (3). Afterward, other 12 unrelated ATTR families with Gly83Arg mutations were reported between 2011 and 2017. Most of these cases were noticed and reported by ophthalmologists. The common features of these patients were predominant ocular involvement in the Chinese Han population. All reported probands had a positive family history, whose family members manifested predominant blurred vision as well. Also, most of these cases were diagnosed by Congo-red staining of the specimens after vitrectomies before being confirmed by *TTR* gene screening. Almost all the cases were early-onset and had the initial symptoms before 50 years old. All the patients initially presented with blurred vision except one patient with onset of CTS (7). Extraocular symptoms were reported only in Liu et al.'s study, which involved one family with 12 patients. In this family, 10 patients had CTS, while only four patients showed the symptoms of sensor-motor neuropathy in lower limbs (7). It seemed that the age of onset was later in women than in men in the same family (6, 12). Only one patient had evidence of cardiac involvement, including atrioventricular block and cardiac hypertrophy (7) (Table 1).

## Discussion

*TTR* Gly83Arg mutation is specific to the Chinese Han population. Chen et al. reported the first Gly83Arg patient in 2008 (3), and then other 12 unrelated Gly83Arg families were consecutively reported between 2011 and 2017 in the Chinese Han population. As Gly83Arg mutation was mainly reported by ophthalmologists, attention had been focused on ocular involvements. Nearly all the patients reported initially presented with VO (3–9, 11–13). Other ocular manifestations included secondary glaucoma (7, 13), cataract (4, 5, 7, 13), retinal vasculopathy (5, 7, 11), xerophthalmia (7), and dyscoria (7). Most patients with Gly83Arg have been reported to have only ocular symptoms so far (3–9, 11–13). In fact, 32 of the 130 (25%) reported *TTR* mutations have been found to have ophthalmic involvement, namely, Val28Met, Leu58Arg, Lys78Phe, Ile84Ser, and so on (15, 16). Of all the patients with ATTR, ocular involvement was found in no more than 10% and usually occurred at an advanced stage (15). Ophthalmic findings included VO, glaucoma, neurotrophic keratitis, and tortuous retinal vessels (16).

The obvious ocular symptoms and a relatively long disease course usually worked together to mask peripheral neuropathy and autonomic dysfunction. However, neuropathy might strike a patient after many years of blurred vision. It was worthy of note that bilateral CTS was likely to occur and could be a vital red flag sign, which seemed like that of Ala97Ser mutation (17, 18). Based on our cohort, heterogeneity of patients with Gly83Arg mutation-related ATTR also existed. Some patients, especially women, predominantly presented with long-term CTS, but male patients had more severe peripheral neuropathy and autonomic dysfunction. However, compared with the common *TTR* gene mutation-related ATTR that usually began with a length-dependent polyneuropathy, developed more severe autonomic dysfunction (16, 19) and could lead to death within 7–12 years of onset if left untreated (1, 20), Gly83Arg mutation-related ATTR had a relatively more benign prognosis and longer survival. Interestingly, male patients in our study had more severe presentations in peripheral neuropathy and autonomic neuropathy than female patients with higher NISs, Norfolk QOL scores, and COMPASS-31 scores and worse results in electrophysiological examinations. Besides, male patients were also found to have a relatively short course of disease with rapid progression. Similarly, Xie et al.'s and Zhang et al.'s studies also revealed the later onset in women than in men in the same family (6, 12). In Liu et al.'s study, all patients with lower extremity involvement were men, and the only patient with heart involvement was also a male patient (7), suggesting that there might be biological factors that inhibited myocardial amyloid infiltration in women or facilitated it in men as in previous observations (21). Therefore, there was still the possibility that Gly83Arg mutation patients could progress to multisystem involvements, especially in male patients, despite a relatively benign course of the disease.

Enlargement of median nerves at the wrist, and proximal enlargement in the median, ulnar, and sciatic nerves were observed by nerve ultrasound, even in asymptomatic carriers, which was consistent with other mutation-related neuropathy of patients with ATTR (10, 22). The CSAs of asymptomatic carriers were smaller than those of symptomatic patients, just as Salvalaggio et al. described (23). These results proved that nerve ultrasound could be a useful tool to identify nerve involvement in patients with Gly83Arg mutation-related ATTR in the early stage of the disease.

Pathologically, for sural nerve biopsy in our study, it was found that the loss of myelinated nerve fiber density did not match the course of neuropathy, that is, the course of the disease was long but the amount of nerve loss was relatively moderate in our study compared with patients with other mutation-related ATTR. It has been reported in previous literature that the myelinated nerve fiber density of patients with early-onset Val30Met mutation-related ATTR decreased by at least 70% after an average course of 3 years (24). However, in patient II-4 with a total course of >4 years, the nerve fiber density decreased only moderately, which offered more evidence for the benign course of Gly83Arg mutation-related neuropathy. In muscle pathology, predominant neurogenic impairment accompanied by possible myogenic impairment was observed, so were TTR amyloid deposits in muscle tissues, indicating that the Gly83Arg mutation type might cause primary muscle damage as previously reported (25).

TTR was mainly synthesized by the liver, choroid plexus of the brain, and the retinal pigment epithelium (RPE) (26). But the ocular symptoms in patients with Gly83Arg mutation predated neurological symptoms. In addition, ocular symptoms in patients with ATTR occurred even after liver transplantation, at which time serum mutant TTR concentrations decreased significantly (27). The predilection of Gly83Arg mutation for fibril formation in the vitreous body was unclear. It could be speculated that the ocular amyloid deposition arose from TTR synthesized by RPE, while the mutant Gly83Arg TTR might be harder to degrade or form larger amyloid deposits within the eye than other *TTR* variants. The mutant TTR might also have a special affinity to the vitreous body (7). An X-ray structure of the TTR tetramer, the EF helix (residues D74–L82), and EF loop (residues G83–E89) played a critical role in maintaining the stability of the TTR tetramer. The mutation of *TTR* Gly83Arg was located in the EF loop region, and embedded the key interface for the TTR-retinol binding protein (RBP) complex formation, which accelerated monomer unfolding and aggregation by destabilizing the EF helix (28, 29). In the study by Liu et al. (7), the subunits of mutant TTR were very close to R62 of RBP, resulting in electrostatic repulsion, seriously compromising the stability of both TTR-RBP. VO could be improved by incomplete vitrectomy (3, 6–9, 11, 13). However, most patients experienced relapse after a few years (7, 8, 13), as was observed in our study. Amyloid continued to be deposited in the residual vitreous in the posterior pole and periphery (7). Therefore, Liu et al. (7) believed that incomplete vitrectomy with posterior vitreous detachment might be a better surgical approach to avoiding a second surgery in patients with this mutation.

According to current standards of care in ATTR, liver transplantation could control the progression of neuropathy (30), especially in early-onset Val30Met mutation types (31). However, the ocular symptoms of patients of the Gly83Arg mutation type tended to occur and aggravate in advance after liver transplantation, which might be related to the fact that RPE continued to produce mutant TTR locally (32). Therefore, it could be speculated that liver transplantation was not an ideal treatment for patients with Gly83Arg mutation. TTR stabilizer, tafamidis, has been proved to slow deterioration of neurological function in patients with Val30Met mutation-related ATTR (33). However, it has also been proved to fail to delay the progression of ocular symptoms in patients with ATTR, possibly because of its inability to cross the blood-brain and blood-retinal barriers (34). The *TTR* gene silencing drugs, RNAi therapy, and antisense oligonucleotides were also effective in phase 3 studies of treatments (35). However, there was no evidence that these drugs could improve ocular symptoms.

In conclusion, Gly83Arg-related ATTR was characterized by early-onset, slow progression, and prominent ocular involvement. But our study recommended extraocular assessment for Gly83Arg-related ATTR of the Chinese Han population. Peripheral nerve symptoms in patients with Gly83Arg started with CTS or a progressive length-dependent peripheral neuropathy. Autonomic symptoms and cardiac involvement could occur. However, heterogeneity of symptoms in different families and genders might also exist. The characteristics of multisystem involvement could help clinicians to make an early diagnosis of ATTR to achieve early detection and early intervention.

## Data Availability Statement

The original contributions presented in the study are included in the article/supplementary material, further inquiries can be directed to the corresponding author.

## Ethics Statement

The studies involving human participants were reviewed and approved by the Clinical Research Ethics Committee of Peking University First Hospital. The patients/participants provided their written informed consent to participate in this study.

## Author Contributions

Acquisition of data, drafting of the initial manuscript, and writing of the final manuscript by ZL. Acquisition of data, data review, and revision of the initial draft by KD. Study concept and design and critical revision of the manuscript by XC, HL, WZ, ZW, and YY. Interpretation of results and revision of the final manuscript by LM. All authors contributed to the article and approved the submitted version.

## Funding

This study was supported by the Beijing Municipal Natural Science Foundation (No. 7194323).

## Conflict of Interest

The authors declare that the research was conducted in the absence of any commercial or financial relationships that could be construed as a potential conflict of interest.

## Publisher's Note

All claims expressed in this article are solely those of the authors and do not necessarily represent those of their affiliated organizations, or those of the publisher, the editors and the reviewers. Any product that may be evaluated in this article, or claim that may be made by its manufacturer, is not guaranteed or endorsed by the publisher.
